# Transcriptome analysis of poplar rust telia reveals overwintering adaptation and tightly coordinated karyogamy and meiosis processes

**DOI:** 10.3389/fpls.2013.00456

**Published:** 2013-11-21

**Authors:** Stéphane Hacquard, Christine Delaruelle, Pascal Frey, Emilie Tisserant, Annegret Kohler, Sébastien Duplessis

**Affiliations:** ^1^INRA, UMR 1136, Interactions Arbres-MicroorganismesChampenoux, France; ^2^UMR 1136, Université de Lorraine, Interactions Arbres-MicroorganismesVandoeuvre-lès-Nancy, France

**Keywords:** *Melampsora larici-populina*, obligate biotrophic fungus, rust lifecycle, teliospores, gene expression, microarray

## Abstract

Most rust fungi have a complex life cycle involving up to five different spore-producing stages. The telial stage that produces melanized overwintering teliospores is one of these and plays a fundamental role for generating genetic diversity as karyogamy and meiosis occur at that stage. Despite the importance of telia for the rust life cycle, almost nothing is known about the fungal genetic programs that are activated in this overwintering structure. In the present study, the transcriptome of telia produced by the poplar rust fungus *Melampsora larici-populina* has been investigated using whole genome exon oligoarrays and RT-qPCR. Comparative expression profiling at the telial and uredinial stages identifies genes specifically expressed or up-regulated in telia including osmotins/thaumatin-like proteins (TLPs) and aquaporins that may reflect specific adaptation to overwintering as well numerous lytic enzymes acting on plant cell wall, reflecting extensive cell wall remodeling at that stage. The temporal dynamics of karyogamy was followed using combined RT-qPCR and DAPI-staining approaches. This reveals that fusion of nuclei and induction of karyogamy-related genes occur simultaneously between the 25 and 39 days post inoculation time frame. Transcript profiling of conserved meiosis genes indicates a preferential induction right after karyogamy and corroborates that meiosis begins prior to overwintering and is interrupted in Meiosis I (prophase I, diplonema stage) until teliospore germination in early spring.

## Introduction

Rust fungi (basidiomycetes, pucciniales) are obligate biotrophs that belong to a monophyletic group containing ~7000 species (Maier et al., 2003). These are one of the most widespread and devastating groups of plant pathogens that can infect monocotyledonous and dicotyledonous plants, including major crop species (Alexopoulos et al., 1996). For example, the emergence of the Ug99 race of the wheat stem rust *Puccinia graminis* f. sp. *tritici* is a serious threat to wheat production worldwide, causing massive crop losses (Singh et al., 2011; Fisher et al., 2012). The genomes of the rust fungi *P. graminis* f. sp *tritici*, infecting wheat and barberry and *Melampsora larici-populina*, infecting poplar and larch, have been sequenced and genome signatures related to their extreme parasitic lifestyle were unraveled (Duplessis et al., 2011a). *M. larici-populina* causes devastating damage on poplar plantations that are used for wood production, carbon sequestration, biofuel production, and phytoremediation (Polle et al., 2013).

Like many other rust fungi, *M. larici-populina* exhibits a complex heteroecious macrocyclic lifecycle completed on two different hosts (poplar, the telial host and larch, the aecial host) and involves five spore-producing stages (Hacquard et al., 2011a). On poplar, the fungus successively differentiates three distinct sporulation structures. The first one, produced throughout spring and summer is called the uredinium and corresponds to a yellow-orange pustule that is differentiated within 7 days on the abaxial surface of poplar leaves. This structure, which releases large amounts of dikaryotic urediniospores, is responsible for massive epidemics in poplar plantations in Europe and worldwide since successive cycles of uredinia formation occur throughout summer (Barrès et al., 2012). On senescent leaves in autumn, the fungus differentiates highly-melanized pustules called telia that produce the overwintering spore form, the dikaryotic teliospores. In spring, teliospores that have undergone karyogamy and meiosis in decaying poplar leaves germinate and produce a new structure called basidium that releases four haploid basidiospores. The basidiospores infect larch needles to form pycnia, which produce haploid pycniospores. After cross-fertilization of pycnia by pycniospores, the fungus forms aecia, which produce dikaryotic aeciospores that infect again poplar leaves. Interestingly, although most of *M. larici-populina* populations undergo host alternation on larch under temperate climates, asexual lineages that overwintered asexually on poplar were recently reported (Xhaard et al., 2011).

Teliospore ontogeny has been described in several rust fungi genera including *Cronartium, Chrysomyxa, Puccinia*, and *Melampsora* (Longo et al., 1979; Moriondo et al., 1989; Mims et al., 1996; Berndt, 1999; Driessen et al., 2005). Maturation of the teliospores is marked by an increase of the cytoplasmic density, an accumulation of lipid droplets and glycogen-like structures, and disappearance of vacuoles (Harder, 1984; Mendgen, 1984). These features may reflect a specific adaptation contributing to teliospore survival during winter. In addition, the presence of chitin in the spore wall was demonstrated using wheat germ agglutinin gold labeling (Mims and Richardson, 2005). Once produced in telia, teliospores undergo karyogamy and meiosis implicating that these spores are an important source of genetic diversity (Schumann and Leonard, 2000). In the rust fungus *P. graminis*, it has been shown that all teliospores have fusion nuclei 42 days post inoculation (dpi) and that meiosis is blocked in prophase I at the diplonema stage when spores enters dormancy (Boehm et al., 1992). Consistent with this, ultrastructure analysis of teliospores revealed that meiotic chromosome pairing (synaptonemal complexes, prophase I) is initiated shortly after karyogamy in *Gymnosporangium* (Mims, 1977, 1981) and that *Puccinia malvacearum* teliospores are in late diplonema stage when they differentiate metabasidia (O'Donnell and McLaughlin, 1981). Taken together, these data suggest that for rust species with overwintering telia, meiosis begins prior to overwintering and is interrupted in Meiosis I (prophase I, diplonema stage) until teliospore germination in early spring.

Despite the importance of the telial stage for the rust life cycle, almost nothing is known about the fungal genetic programs that are activated in this overwintering structure. Indeed, most of the recent molecular approaches to understand rust biology have focused on the analysis of gene expression in urediniospores and during host infection at the uredinial stage using Sanger EST sequencing (for a complete list, see recent review by Duplessis et al., 2012), microarrays (Jakupović et al., 2006; Hacquard et al., 2010; Duplessis et al., 2011a,b) or RNA-Seq (Fernandez et al., 2012; Petre et al., 2012; Cantu et al., 2013; Garnica et al., 2013). Recently however, ESTs libraries generated from different spore types of the wheat leaf rust fungus *Puccinia triticina* revealed a high proportion of EST sequences (87% of 697 ESTs) uniquely detected in teliospores compared to all other sampled stages (Xu et al., 2011).

In the present study, we used whole-genome custom oligoarrays to monitor fungal gene expression profiles in telia of *M. larici-populina* collected on senescent poplar leaves before overwintering. Comparative expression profiling at the telial and uredinial stages identifies genes that are only or preferentially expressed in telia, suggesting their contribution to a specific genetic program. We further investigated some candidate genes that might be involved in the teliospore differentiation process.

## Materials and methods

### Plant growth conditions and inoculation procedures

Samples corresponding to resting urediniospores (USP), infected poplar leaves (INF; 96 h post inoculation, hpi), and uredinia (URE; 168 hpi) were previously described (Duplessis et al., 2011b). For microarray analysis, senescent leaves of the “Beaupré” poplar cultivar naturally infected by *M. larici-populina* and presenting dark telial pustules (telia, TEL) were harvested in October 2010 at a poplar nursery (Centre INRA Nancy Lorraine, Champenoux, 54, France). For RT-qPCR and karyogamy process analyses, development of *M. larici-populina* (strain 98AG31) telia was monitored in the susceptible poplar cultivar *Populus trichocarpa* × *Populus deltoides* “Beaupré” (compatible interaction). Resting urediniospores were collected on leaves of susceptible *P. deltoides* × *Populus nigra* “Robusta” and plant inoculation procedures were performed using the same inoculum dose of 100,000 urediniospores/ml and strictly identical culture conditions as those previously described (Rinaldi et al., 2007). Samples were harvested at intervals corresponding to the biotrophic growth (4 days post inoculation, dpi), the formation of uredinia (11 dpi), and the formation and the maturation of telia (18, 25, 32, 39, and 46 dpi). Infected leaves were incubated at 20°C until uredinia formation (11 dpi) and then transferred and maintained at 10°C to induce telia formation. At each time-point, harvested samples were immediately fixed in 4% (wt/vol) paraformaldehyde (PFA) for microscopy analyses or snap frozen in liquid nitrogen and kept at −80°C for further nucleic acid isolation. The time course was performed in triplicate.

### Microscopy

After fixation (3 h, 4°C) in 4% PFA (wt/vol) prepared in phosphate buffer saline (PBS), samples were washed twice with PBS and then embedded in 6% agarose (wt/vol). Transversal sections (15 μm) of INF were cut using a vibratome VT1000S (Leica, Nanterre, France) and directly transferred onto a microscopic slide. Sections were mounted in an antifade reagent with DAPI (Molecular Probes) and observed using the Palm Laser Micro dissection Microscope (Zeiss, Bernried, Germany) using the 40× objective. The number of fused and non-fused nuclei were analyzed for each sample in ~100 teliospores from a single biological replicate.

### RNA isolation and cDNA synthesis

Total RNA were isolated with the RNeasy Plant Mini kit (Qiagen, Courtaboeuf, France) from 1 mg of resting spores (USP) and from 100 mg of infected leaf tissues (INF, URE) as previously described (Duplessis et al., 2011b), including a DNase I (Qiagen) treatment according to the manufacturer's instructions to eliminate traces of genomic DNA. Total RNA from the telial stage (TEL) were isolated from 100 mg of leaf tissue using the same protocol used for the USP, INF, and URE samples. Electrophoretic RNA profiles were assessed with an Experion analyzer using the Experion RNA Standard-sens analysis kit (Bio-Rad, Marnes la Coquette, France) (Figure S1). For oligoarrays experiment, total RNA from the telial stage (TEL) were subjected to a single round of amplification using the MessageAmp™ II aRNA amplification kit (Ambion, Austin, TX, USA) as previously described for the USP, INF et URE samples (Duplessis et al., 2011b). RNA amplification generated more than 50 μg of amplified RNA (aRNA) and aRNA profiles were verified using the Experion analyzer and Experion RNA Standard-Sens analysis kit (Bio-Rad). Double-stranded cDNA were synthesized from 2.5 μg of aRNA using the Superscript™ Double-Stranded cDNA Synthesis Kit (Invitrogen, Cergy Pontoise, France) according to the NimbleGen user protocol. Single dye labeling of samples, hybridization procedures, and data acquisition were performed at the NimbleGen facilities (NimbleGen Systems, Reykjavik, Iceland) following their standard protocol. For the RT-qPCR analysis, isolation of total RNA was performed using the RNeasy Plant Mini kit (Qiagen, Courtaboeuf, France) from 50 mg of infected leaf tissues (4–46 dpi) and a DNase I treatment was included to eliminate traces of genomic DNA (Qiagen). Electrophoretic RNA profiles were assessed with an Experion Analyzer using the Experion RNA Standard-sens analysis kit (Bio-Rad, Marnes la Coquette, France) (Figure S1).

### Construction of *m. larici-populina* exon oligoarray

The *M. larici-populina* custom-exon expression oligoarray (4 × 72 K) manufactured by Roche NimbleGen Systems Limited (Madison, WI) (http://www.nimblegen.com/products/exp/index.html) contained four independent, non-identical, 60-*mer* probes per gene model coding sequence (NCBI Gene Expression Omnibus, GEO platform GPL10350). Included in the oligoarray were 17,556 coding sequences, 1063 random 60-*mer* control probes and labeling controls (Duplessis et al., 2011a). The 17,556 coding sequences correspond to the initial *M. larici-populina* gene annotation set at the Joint Genome Institute (JGI). The current annotation of the *M. larici-populina* genome is of 16,400 genes (12/06/2013), of which 13,093 (80%) were represented on the array used in the study. Oligonucleotide probes that presented a risk of cross-hybridization with poplar transcripts (i.e., fluorescence signal over the background level when arrays were hybridized with non-inoculated poplar leaf cDNA) or between transcript species expressed by different genes from a same gene family (i.e., probes with more than 90% homology between two transcripts) were not considered in our analysis.

### Microarray data analysis

Microarray probe intensities were quantile normalized across chips. Average expression levels were calculated for each gene from the independent probes on the array and were used for further analysis. Raw array data were normalized using the ARRAYSTAR software (DNASTAR, Inc. Madison, WI, USA). A transcript was deemed expressed when its signal intensity was three-fold higher than the mean signal-to-noise threshold (cut-off value) of 1063 random oligonucleotide probes present on the array. All expression assays were conducted on three independent biological replicates. A Student *t*-test with false discovery rate (FDR) (Benjamini-Hochberg) multiple testing correction was applied to the data (ARRAYSTAR software). Transcripts with a significant *p*-value (<0.05) and more than a 2-fold change in transcript level were considered as differentially expressed. The expression datasets are available at the NCBI GEO as serie #GSE49099.

### Heatmaps of gene expression profiles

Heatmaps of *M. larici-populina* gene expression profiles were generated using the Genesis expression analysis package (Sturn et al., 2002). To derive expression patterns of genes in the different fungal developmental stages (USP, INF, URE, TEL), log2 expression ratios (Relative Expression Indexes, REI) were calculated between the normalized expression level for a given gene at a given fungal developmental stage and the geometrical mean expression level calculated across all 4 fungal developmental stages (Duplessis et al., 2011b). Functional gene annotation was based on Blastp search against the Swissprot database (Bairoch and Apweiler, 2000).

### KOG enrichment analysis

We obtained KOG (eukaryotic orthologous groups) (Tatusov et al., 2003) annotation of each *M. larici-populina* gene by using RPSBLAST against the KOG database (*e*-value < 1e-5). Each gene was classified according to KOG functional classification using custom perl scripts. Over-represented KOG categories among telia- or uredinia-induced genes were calculated relative to the global gene distribution. The significance of over-represented functional KOG categories were evaluated using the Fisher's Exact Test (*p* < 0.05).

### RT-qPCR

To monitor karyogamy- and meiosis-related transcript expression profiles during telia formation, 16 genes were selected for RT-qPCR assays (Table S1). Specific primers amplifying fragments ranging from 152 to 247 were designed for each gene using Primer 3 (Rozen and Skaletsky, 2000). Absence of cross annealing was checked in the *M. larici-populina* (http://genome.jgi-psf.org/Mellp1/Mellp1.home.html) and *P. trichocarpa* (http://www.phytozome.net/) genome sequences using the blastn algorithm. First-strand cDNA synthesis was performed using 500 ng total RNA and cDNA were amplified strictly following procedures described in Hacquard et al. (2012). Transcript expression levels were normalized with the *M. larici-populina* reference genes a-tubulin (*Mlp-aTUB*) and elongation factor (*Mlp-ELF1*a) as previously described (Hacquard et al., 2011b).

## Results

### Development of uredinia and telia within poplar leaves

**Figure 1:**
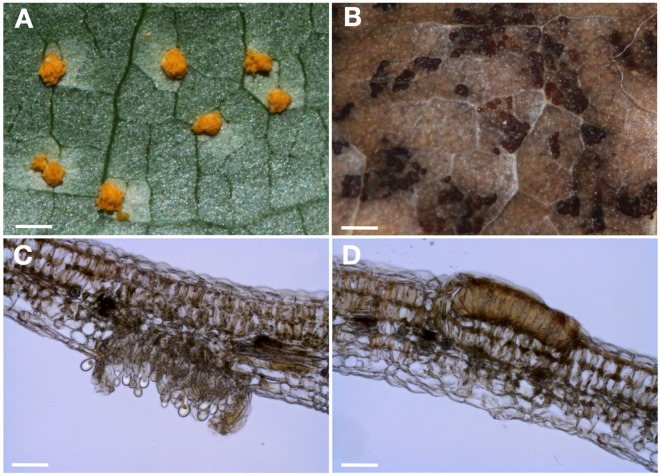
***M. larici-populina* uredinia and telia on poplar leaves. (A)** Macroscopic picture of uredinia formed 7 days post inoculation by the virulent *M. larici-populina* 98AG31 strain on the abaxial surface of leaves of the “Beaupré” poplar cultivar. **(B)** Macroscopic picture of telia observed early in autumn on the adaxial surface of senescent “Beaupré” leaves naturally infected by *M. larici-populina*. Scale bar = 1 mm. **(C)** Transversal section of a “Beaupré” leaf infected by the virulent *M. larici-populina* 98AG31 strain showing a mature uredinium releasing urediniospores on the abaxial surface. **(D)** Transversal section of a “Beaupré” leaf naturally infected by *M. larici-populina* showing a mature telia on the adaxial surface into which teliospores are tightly encapsulated. Scale bar = 50μm.

Uredinia and telia that are formed by the rust fungus *M. larici-populina* on the susceptible poplar cultivar “Beaupré” are represented in Figure 1. During summer, yellow-orange uredinia pustules are formed on the abaxial surface of poplar leaves about 1 week after urediniospore landing on poplar leaf epidermis (Figure 1A). Early in autumn, the asexual uredinial cycle stops and black telia pustules (Figure 1B) start to differentiate on the adaxial surface of poplar leaves. Whereas uredinia continuously release important amounts of urediniospores that are dispersed over large distances by wind to cyclically infect poplar throughout summer (Figure 1C), teliospores are produced only once a year in telia and those are tightly encapsulated between the plant epidermis and the palisade mesophyll of poplar leaves (Figure 1D). This structure provides adequate conditions for teliospore overwintering.

### *M. larici-populina* gene expression profiling in telia and comparison with the uredinial stage

**Figure 2:**
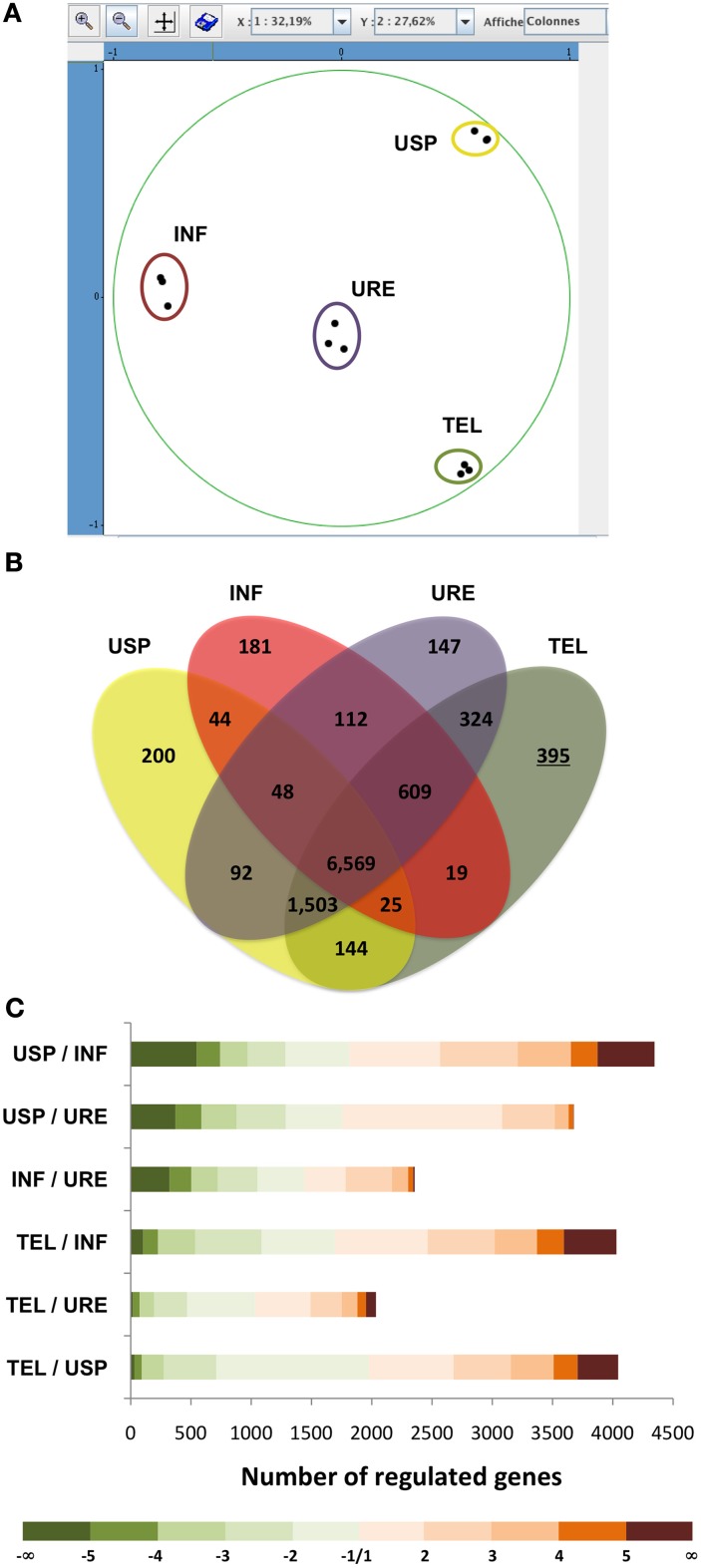
**Microarray expression analysis of telia and comparison with expression data of other fungal developmental stages. (A)** Principal component analysis (PCA) of *M. larici-populina* transcript levels measured in urediniospores (USP), during infection (INF), in uredinia (URE), and in telia (TEL) using custom oligoarrays (three biological replicates per stage were used for the PCA). Expression level of each gene assessed in a given biological situation and a given biological replicate was reported to the mean expression level calculated for the 12 hybridizations (six conditions × three replicates) and was log_10_-normalized before proceeding with PCA. The PCA plot places biological conditions along the two axes (X and Y) explaining 32.19 and 27.62% of the variance observed within samples. **(B)** Venn diagram showing the number of *M. larici-populina* genes expressed in each condition. The underlined number corresponds to the number of genes specifically expressed in telia. **(C)** Number of genes significantly regulated between the analyzed fungal developmental stages [log_2_ Fold-Change (FC) >1, *p* < 0.05]. Color coding corresponds to log_2_FC.

To identify *M. larici-populina* genes significantly regulated (*p*-value <0.05; −2 < fold change > 2) or specifically expressed at the telial stage, we used a whole-genome custom oligoarray onto which oligonucleotides matching to 13,093 genes of *M. larici-populina* were spotted (Duplessis et al., 2011a). We compared transcript levels measured in telia (TEL) with those detected in three previously described samples related to the uredinial stage (urediniospores: USP, infection process: INF, uredinia: URE) (Duplessis et al., 2011b). Principal component analysis of transcript expression levels measured in USP, INF, URE, and TEL showed very good consistency between the three biological replicates of collected telia (Figure 2A). Furthermore, it appears clearly that distinct genetic programs are expressed by the rust fungus at the four developmental stages surveyed. USP, INF, and TEL samples are distributed apart by the PCA and URE samples have an intermediate position, which could suggest overlapping expression patterns between the different types of samples (Figure 2A).

### Genes specifically expressed in telia

Among the 13,093 genes present on the oligoarray, 10,412 were expressed in at least one of the four considered developmental stages (Figure 2B, Table S2). Among those, 6569 were expressed in all four fungal developmental stages and 9588 were expressed in telia including 395 telia-specific genes. Interestingly, this is the largest number of specific transcripts found for a given stage since only 200 were detected for USP, 181 for INF, and 147 for URE suggesting that specific functions are activated in the *M. larici-populina* overwintering spore-producing structure. Table 1 summarizes the set of telia-specific genes showing high level of transcript accumulation at that stage (gene expression level >800). Interestingly, a gene encoding a saccharopine dehydrogenase, involved in the biosynthesis of the amino acid L-Lysine through the α-aminoadipate pathway (Xu et al., 2006), ranged among the most highly expressed telia-specific gene set (Expression level >10,000, Table 1). The high proportion of genes encoding unknown proteins (47/68), including 11 Small Secreted Proteins (SSPs), suggests that telia formation and teliospores production is mostly driven by complex and largely unknown mechanisms. Nevertheless, genes encoding several carbohydrate Active Enzymes (CAZymes, Cantarel et al., 2009) including a pectinesterase and a cutinase (*Mlp-93655, Mlp-123715*) (Carbohydrate esterase families 8 and 5, respectively), an alpha-1,6-mannosyltransferase (*Mlp-27594*, glycosyl transferase family 32) and an endoglucanase (*Mlp-95634*, glycolsyl hydrolase family 12) could be identified (Table 1). Consistent with the fact that karyogamy and meiosis occur during teliospore maturation, several meiotic and karyogamy related genes were also specifically expressed in the telial structure (Table 1) such as those encoding the meiotic recombination protein rec8, the cell division control protein 15, the meiotic nuclear division protein 1 as well as the nuclear fusion protein Kar5 and Kar9 (*Mlp-93153, Mlp-94329, Mlp-106571, Mlp-112713, Mlp-94206*).

**Table 1:** ***M. larici-populina* genes highly and specifically expressed in telia**.

**Protein_ID^a^**	**Expression level^b^**				**Definition**	**Cat^c^**	**Length^d^**	**SP^e^**	**Blastp *Pgt*^f^**
	**USP**	**INF**	**URE**	**TEL**					
86396	87	47	76	14,466	Hypothetical protein		184		PGTG_08052
101624	28	27	27	12,711	Saccharopine dehydrogenase	A	434		PGTG_03759
87547	38	22	25	11,939	Hypothetical SSP, PR-1-like protein	B	280	Y	PGTG_07743
101938	60	41	33	11,876	Hypothetical SSP	B	133	Y	–
93655	26	77	45	11,548	Pectinesterase (CE8)	C	316	Y	PGTG_08012
36325	102	28	51	10,214	Homogentisate 1,2-dioxygenase	A	414		–
107118	22	22	23	9702	Hypothetical SSP, PR-1-like protein	B	236	Y	PGTG_16765
27934	34	28	28	8747	Glycosyl transferase (GT8)	C	188		–
88840	65	157	66	7067	Hypothetical protein		519		PGTG_11713
27594	59	53	44	7029	Alpha-1,6-mannosyltransferase (GT32)	C	249		–
58538	32	31	90	6606	Short-chain dehydrogenase	A	248		PGTG_15283
104079	26	23	37	6478	Hypothetical protein		337		PGTG_03998
110542	34	97	49	5369	Hypothetical SSP	B	133		–
95634	36	38	29	5193	Endoglucanase (GH12)	C	179		PGTG_03891
108357	24	61	69	4899	Hypothetical protein		508		PGTG_16954
110660	69	38	41	4587	Hypothetical protein		513		PGTG_15396
92814	26	22	24	4576	Hypothetical protein		768		PGTG_06712
93153	34	34	44	4305	Meiotic recombination protein rec8	D	774		PGTG_02404
87054	71	66	80	4229	Hypothetical protein		279		PGTG_13349
59662	30	21	23	3898	Hypothetical protein		326		–
93477	25	38	27	3199	Hypothetical protein		481		PGTG_02434
112713	67	38	76	3023	Nuclear fusion protein KAR5	D	736		PGTG_16428
101708	31	146	68	2622	Aldehyde dehydrogenase	A	496		PGTG_15008
104617	29	34	39	2541	Hypothetical protein		308		–
86447	34	30	23	2401	Hypothetical protein		573		PGTG_18083
92775	28	32	28	2392	Hypothetical protein		269		–
109764	76	102	62	2269	Hypothetical protein		482		–
85892	48	31	30	2239	Hypothetical protein		402		PGTG_13349
84888	30	34	51	2146	Hypothetical protein		626		PGTG_00821
92678	60	74	30	2140	Hypothetical protein		419		PGTG_01636
123561	32	31	76	2077	Hypothetical SSP	B	133	Y	–
103910	51	71	37	2072	Hypothetical protein		455		PGTG_10254
62289	45	45	56	1924	Hypothetical SSP	B	265	Y	PGTG_06969
61331	63	26	41	1883	Hypothetical protein		197		–
26257	102	38	48	1842	Aquaporin (MIP)	E	263		PGTG_02867
89049	45	54	47	1767	Hypothetical protein		447		PGTG_03343
60216	34	26	49	1702	Hypothetical SSP	B	214	Y	–
94329	26	37	51	1573	Cell division control protein 15	D	451		PGTG_16937
59440	70	33	31	1553	Hypothetical protein		336		PGTG_09936
113347	44	31	59	1536	Hypothetical SSP	B	136	Y	–
123715	44	81	80	1520	Cutinase (CE5)	C	351	Y	PGTG_01091
109924	29	24	18	1449	Hypothetical protein		297		–
110784	69	56	33	1442	Hypothetical protein		518		PGTG_15984
66126	29	28	22	1426	Carbohydrate esterase (CE16)	C	262		PGTG_18191
106798	23	39	31	1419	Hypothetical secreted protein		325		–
101151	27	19	19	1362	Choline dehydrogenase	A	594		PGTG_18542
110516	42	41	67	1336	Hypothetical protein		687	Y	–
90260	83	41	42	1279	Homeobox protein		116		PGTG_07066
64744	108	218	83	1274	Hypothetical protein		290		–
91904	102	227	69	1269	Hypothetical protein		363		–
93339	31	26	32	1249	Hypothetical protein		435		PGTG_01619
90367	49	54	41	1216	Hypothetical protein		589		PGTG_04717
103627	40	18	26	1192	Hypothetical protein		167		–
102200	36	29	83	1174	Hypothetical SSP	B	153	Y	–
61074	29	43	66	1124	Hypothetical protein		454		PGTG_03998
63861	31	27	28	1105	Hypothetical protein		485		–
65250	20	22	19	1071	Hypothetical SSP	B	195	Y	PGTG_06052
70334	44	35	26	1061	Hypothetical protein		109		–
113400	45	46	31	1011	Hypothetical protein		498		PGTG_19950
63565	78	201	99	989	Hypothetical protein		176		PGTG_11018
106571	84	33	42	870	Meiotic nuclear division protein 1	D	206		PGTG_18915
25325	33	57	33	869	L-gulonolactone oxidase	A	442		PGTG_07192
101135	106	59	50	866	Hypothetical protein		220		–
94206	108	95	53	850	hypothetical protein (distantly related to KAR9)	D	656	Y	–
107936	45	55	75	821	Hypothetical SSP	B	160	Y	–
105800	55	45	73	810	Hypothetical protein		342		PGTG_1213

a Protein ID number of corresponding best gene model in the M. larici-populina JGI genome sequence.

b Normalized transcript levels are presented. Transcripts levels in urediniospores (USP), infection process (INF), and uredinia (URE) samples are below our arbitrary cut-off (less than three-fold higher than the mean signal-to-noise threshold) and were thus considered as not expressed. TEL, Telia.

c Cat, Category: A, metabolism enzymes; B, small secreted proteins (SSPs); C, carbohydrate-active enzymes; D, meiosis and karyogamy-related genes; E, transporters.

d Protein length (amino acids).

e SP, Signal Peptide. SPs were predicted according to SignalP v3.0.

f Pgt, Puccinia graminis f. sp. tritici (http://www.broadinstitute.org/annotation/genome/puccinia_group/MultiHome.html).

### Genes regulated in telia compared with other *m. larici-populina* developmental stages

By comparing transcript expression levels in TEL and URE, we identified 2035 genes significantly regulated (−2 < fold-change >2, *p*-value < 0.05) between the two spore-producing structures (Figure 2C, Table S2). A relatively similar set of regulated genes was detected between the INF and URE conditions (<2500) but larger numbers were detected between USP and INF, USP, and URE, TEL and INF, or TEL and USP (>3600) (Figure 2C). This result is in accordance with the principal component analysis (Figure 2A) and supports the idea that similar sets of genes are shared by the genetic programs triggered in the rust fungus *M. larici-populina* for the production of both telia and uredinia. Despite potential common features, the substantial number of genes identified as significantly regulated in TEL compared with URE (1003 up- and 1032 down-regulated genes) indicates that specific pathways may be activated and could explain the structural and functional differences characterizing the two structures (Figure 2C).

### Genes up-regulated in telia compared with uredinia

**Figure 3:**
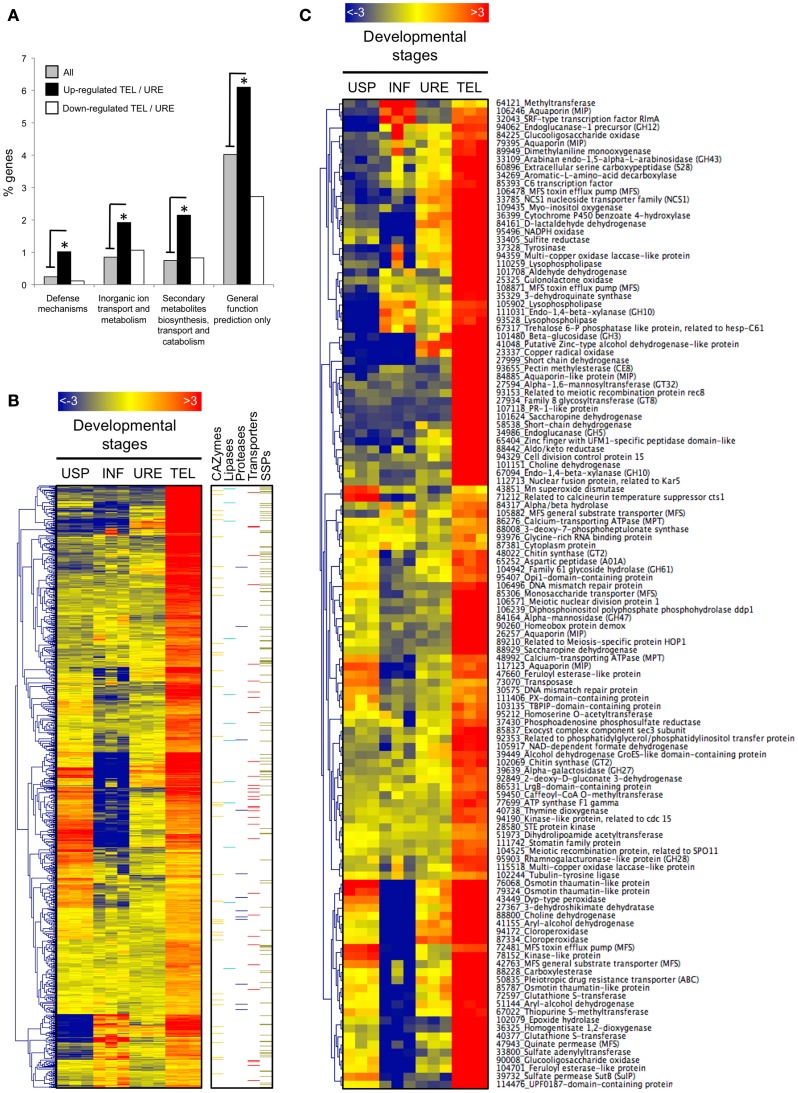
**Genes significantly up-regulated in telia compared with uredinia. (A)** Over-represented KOG categories among telia-induced genes relative to the global gene distribution. Black and white bars correspond to the distribution of genes significantly up and down regulated in telia (TEL) compared with uredinia (URE) (log_2_FC >1, *p* < 0.05), respectively, into functional KOG categories. Gray bars correspond to the global gene distribution. Only the significantly over-represented functional KOG categories are presented. ^*^indicate statistically significant differences (Fisher’s Exact Test, *p* < 0.05) **(B)** Heatmap of transcript expression levels in all four fungal developmental stages for genes significantly up-regulated in telia compared with uredinia (log_2_FC >1, *p* < 0.05). Over-represented (red) or under-represented (blue) trancripts are depicted as log_2_ fold-changes relative to the mean expression level measured across all four stages. USP, urediniospores; INF, poplar infected leaves; URE, uredinia; TEL, telia. On the right side, genes belonging to five pathogenicity-related categories (carbohydrate active-enzymes, lipases, proteases, transporters, and small secreted proteins) are highlighted with color bars. **(C)** Among the genes presented in the panel **(B)**, only those showing a higher transcript induction in TEL compared with URE (log_2_FC >2, *p* < 0.05) and having a functional annotation (based on the swissprot database) are highlighted. JGI protein identification number and the associated function are indicated.

A functional KOG analysis of the 1003 significantly up-regulated genes in TEL compared with URE reveals significant enrichment for gene categories related to defense mechanism, inorganic ion transport and metabolism, secondary metabolites biosynthesis transport and catabolism as well as general function (Figure 3A). Global expression profiling of these up-regulated genes across all stages revealed that most transcripts significantly accumulated in TEL compared with URE are preferentially expressed in telia except for a cluster of genes that also show high transcripts accumulation in USP (Figure 3B). Interestingly, genes belonging to this cluster are not or barely detected during infection (INF) and several encode transporters (Figure 3B), including a calcium-transporting ATPase (*Mlp-86276*), an aquaporin (*Mlp-26257*), an MFS efflux pump (*Mlp-72481*), an MFS general substrate transporter (*Mlp-42763*), a pleiotropic drug resistance transporter (*Mlp-50834*), a quinate permease (*Mlp-47943*), and a sulfate permease (*Mlp-39732*) (Figure 3C). A total of 113 genes encoding SSPs of unknown function previously categorized as putative candidate effectors (Hacquard et al., 2012) are induced in TEL compared with URE (Figure 3B). Considering the development of telia on senescent leaf tissues, these are most likely not effectors engaged in the manipulation of host cell immunity (Win et al., 2012). Alternatively, they may also have dual functions during the rust lifecycle such as recently reported for the rust transferred protein 1. In addition to its ability to be transferred in the host cytoplasm (Kemen et al., 2005), RTP1 is also capable of fibril formation (Kemen et al., 2013). Thus, some candidate effectors may also have a structural role during teliospores production and maturation. Investigation of the set of significantly regulated genes (fold-change >4) by a functional annotation based on Blastp search against the Swissprot database highlights major biological processes induced during telia formation (Figure 3C). Several genes potentially related to the overwintering process were identified including 5 aquaporins (*Mlp-106246, Mlp-79395, Mlp-84885, Mlp-26257, Mlp-117123*), 3 osmotin/thaumatin-like proteins (TLPs; *Mlp-76068, Mlp-79324, Mlp-85787*), a trehalose-like protein (*Mlp-67317*), a calcineurin temperature suppressor (*Mlp-71212*), and a calcium-transporting ATPase (*Mlp-48992*). Consistent with the functional KOG analysis (Figure 3A), secondary metabolites transport appears to be active in telia since transcripts encoding 3 MFS toxin efflux pumps (*Mlp-106478, Mlp-108871, Mlp-72481*) and a pleiotropic drug resistance transporter (*Mlp-50835*) were strongly accumulated. In addition to the four afore-mentioned telia-specific karyogamy and meiosis-related genes (Table 1), the meiosis-specific protein HOP1, a kinase-like protein (related to cdc15), and the meiotic recombination protein SPO11 are also regulated between TEL and URE, with higher transcript levels detected in TEL. A significant number of genes (8 in total) encoding plant cell wall degrading enzymes dominate among the most highly up-regulated genes in telia supporting an extensive plant cell wall remodeling during telia development. These include CAZymes targeting cellulose (GH12 and GH61 families), hemicellulose (GH10 and GH27 families), pectin (CE8 and GH28 families), and hemicellulose/pectin (GH43 family). Genes encoding two multi-copper oxidase laccase-like proteins, previously detected in a *P. triticina* teliospores EST library (Xu et al., 2011), were also strongly up-regulated in the overwintering telial structure.

### Genes down-regulated in telia compared with uredinia

**Figure 4:**
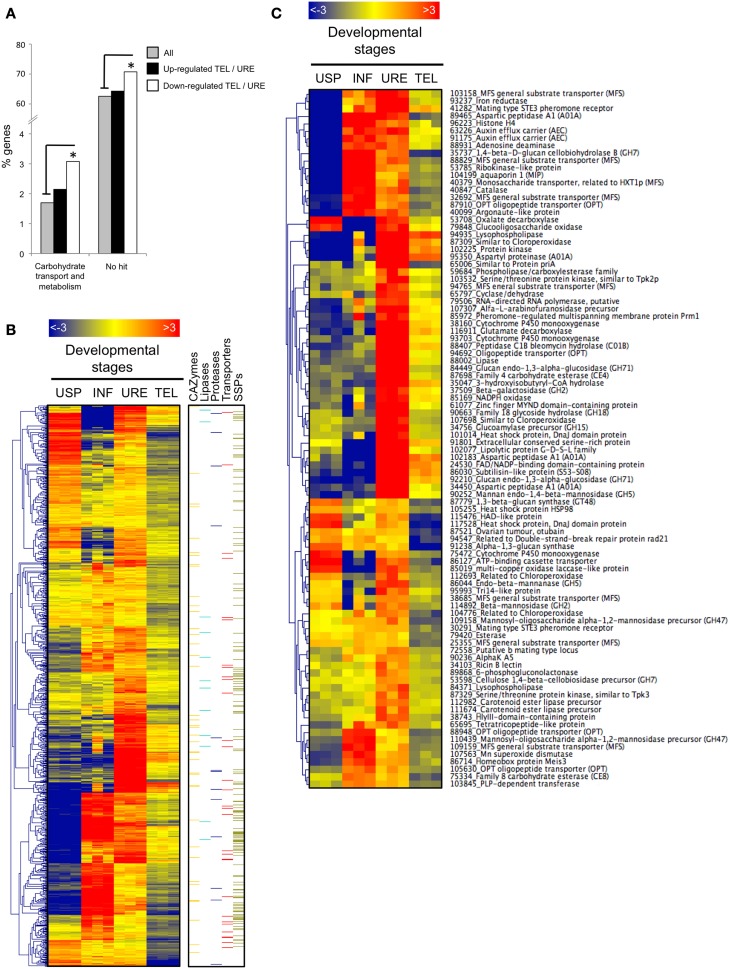
**Genes significantly down-regulated in telia compared with uredinia. (A)** Over-represented KOG categories among telia-repressed genes relative to the global gene distribution. Black and white bars correspond to the distribution of genes significantly up and down regulated in telia (TEL) compared with uredinia (URE) (log_2_FC >1, *p* < 0.05), respectively, into functional KOG categories. Gray bars correspond to the global gene distribution. Only the significantly over-represented functional KOG categories are presented. ^*^indicate statistically significant differences (Fisher’s Exact Test, *p* < 0.05). Heatmap of transcript expression levels in all four fungal developmental stages for genes significantly down-regulated in telia compared with uredinia (log_2_FC < -1, *p* < 0.05). Over-represented (red) or under-represented (blue) transcripts are depicted as log_2_ fold-changes relative to the mean expression level measured across all four stages. USP, urediniospores; INF, poplar infected leaves; URE, uredinia; TEL, telia. On the right side, genes belonging to five pathogenicity-related categories (carbohydrate active-enzymes, lipases, proteases, transporters, and small secreted proteins) are highlighted with color bars. **(C)** Among the genes presented in the panel **(B)**, only those showing high transcript repression in telia compared with uredinia (log_2_FC < -2, *p* < 0.05) and having a functional annotation (based on the swissprot database) are highlighted. JGI protein identification number and the associated function are indicated.

A functional KOG analysis of the 1032 significantly down-regulated genes in TEL compared with URE reveals a significant enrichment for gene categories related to carbohydrate transport and metabolism and unknown proteins (Figure 4A). Transcript profiling of these down-regulated genes revealed that most are preferentially expressed in uredinia but a subset also shows a higher transcript accumulation in USP or during the infection process (INF) (Figure 4B). Importantly, the cluster of genes showing high transcript accumulation during the infection process is particularly enriched with SSPs suggesting they may encode biotrophy-associated effectors involved in poplar immunity manipulation (Figure 4B). By looking at the functional annotation of significantly regulated genes (fold-change < −4), we could identify processes that are activated in uredinia and switched-off in telia (Figure 4C). Among these, we identified 4 genes encoding mating-related proteins including two mating-type STE3 pheromone receptors, a pheromone-regulated multispanning membrane protein (Prm1) and a putative b mating type protein. Consistent with the high content of carotenoid in the urediniospores, two genes encoding carotenoid ester lipase precursors were also up-regulated during urediniospore production and release. Among the genes encoding transporters that are down-regulated in telia compared to uredinia (fold change < −4), we identified six MFS general substrate transporters, four oligopeptides transporters, two auxin efflux carriers, a monosaccharide transporter (related to *Uromyces fabae* HXT1, Voegele et al., 2001) and an aquaporin (Figure 4C). In addition, 14 genes encoding CAZymes and targeting the plant cell wall (GH7, CE8), the fungal cell wall (GH18, GH71), or both (GH2, GH5) showed altered transcript accumulation in TEL compared with URE suggesting that these genes are involved in urediniospore production, maturation processes, or release from host tissues (Figure 4C).

### Expression profiling of karyogamy and meiosis-related genes during telia formation

**Figure 5:**
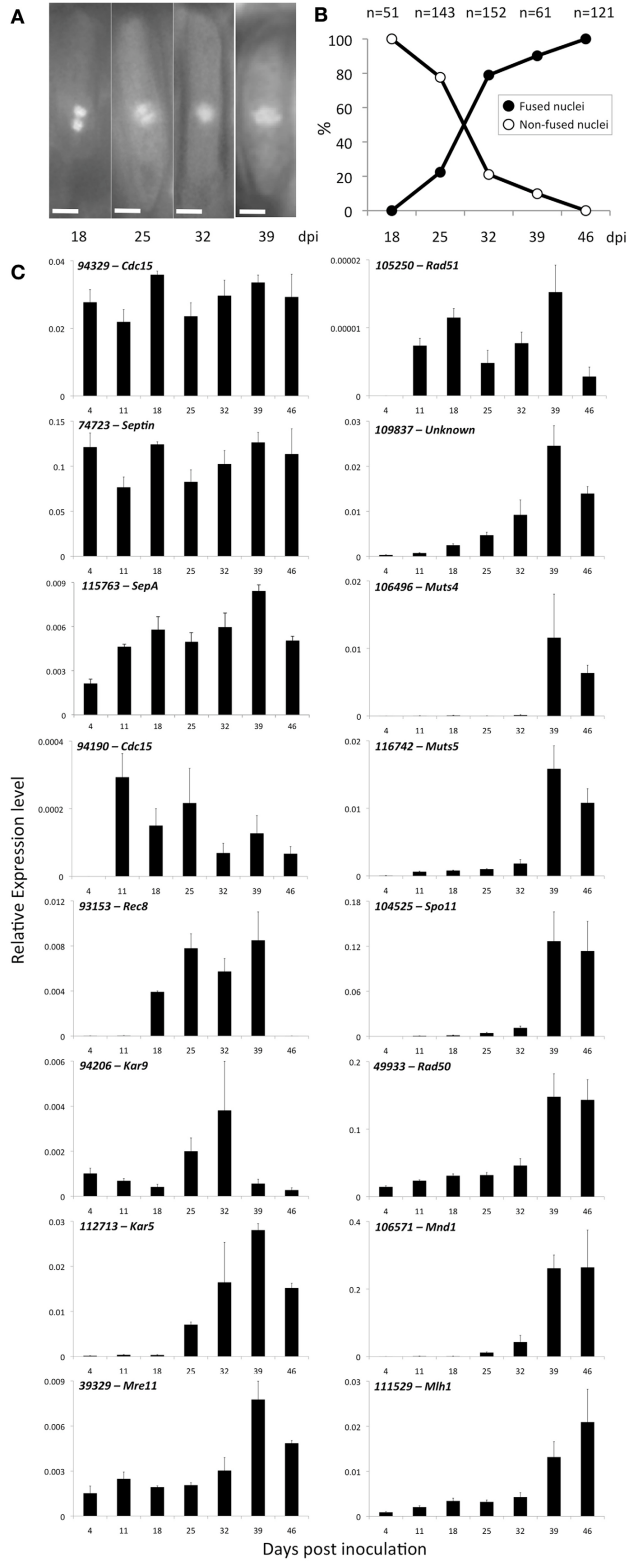
**Karyogamy dynamics and meiotic-related gene expression profiles during telia formation. (A)** Representative pictures of DAPI-stained teliospores nuclei during telia formation and maturation. From left to right, 18, 25, 32, and 39 days post inoculation. Scale bar = 5μm. **(B)** Dynamics of karyogamy during telia formation and maturation. Percentage of fused and non-fused nuclei is indicated for each stage and the total numbers of counted teliospores are indicated above the graph. **(C)** Karyogamy, meiosis, and cytokinesis-related gene expression profiles monitored by RT-qPCR during telia formation and maturation. For each gene, expression levels were normalized with a-tubulin (*Mlp-aTUB*) and elongation factor (*Mlp-ELF1*a) reference genes.

To determine the temporal dynamics of karyogamy and meiosis-related gene expression profiles during telia formation, a time-course interaction survey has been carried out between the *M. larici-populina* virulent strain 98AG31 and detached leaves of the susceptible poplar cultivar “Beaupré.” Samples were harvested at intervals corresponding to the biotrophic growth phase (4 dpi), uredinia (11 dpi), and the formation and the maturation of telia (18, 25, 32, 39, and 46 dpi). Transversal sections of infected leaf tissues followed by DAPI-staining revealed that the first aggregates of fungal cells, corresponding to telia initials, are formed between 14 and 18 dpi. At 18 dpi all observed non-mature teliospores were dikaryotic (Figures 5A,B). The number of fused nuclei increase dramatically from 20% at 25 dpi to 80% at 32 dpi, indicating that karyogamy mainly takes place during this time frame (Figures 5A,B). Consistent with this, RT-qPCR expression profiling of the karyogamy-related genes *Kar5* and *Kar9* revealed that both genes are induced as soon as 25 dpi and their transcripts strongly accumulate at 32 dpi for *Kar9* and 39 dpi for *Kar5* (Figure 5C). All observed teliospores have fused nuclei at 46 dpi (Figures 5A,B). Importantly, all the conserved eukaryotic meiotic genes analyzed in this study (*Rec8, Mre11, Rad50, Rad51, MutS4, MutS5, Spo11, Mnd1*, and *Mlh1;* Malik et al., 2008) are induced in differentiated telia and their transcripts predominantly accumulate at late stages (i.e., 39 and 46 dpi, Figure 5C). This result suggests that with our experimental conditions, meiosis is initiated soon after karyogamy since at 39 dpi, more than 90% of the observed teliospores already contain fused nuclei (Figure 5B). Rad51, essential for double-strand break meiotic repair and Rec8, involved in sister chromatin cohesion, show a particular expression pattern with sustained transcript accumulation throughout the telia differentiation process, except at 46dpi where the transcripts were barely detected. This result may indicate that Rad51 and Rec8 are already produced during karyogamy, before meiosis takes place.

## Discussion

The telial stage of rust fungi plays a crucial role in the fungal life cycle as it produces overwintering spores (i.e., teliospores) in which karyogamy and meiosis take place. Numerous ultrastructural studies have been conducted on telia and teliospores (Longo et al., 1979; Mendgen, 1984; Moriondo et al., 1989; Mims et al., 1996; Berndt, 1999; Driessen et al., 2005; Mims and Richardson, 2005), however, the functions activated by rust fungi during teliospores production and maturation remain poorly described. In the present study, we report in the poplar-poplar rust model pathosystem (Duplessis et al., 2009; Hacquard et al., 2011a) the transcriptome of telia produced by the rust fungus *M. larici-populina* using whole genome exon oligoarrays and RT-qPCR.

Our data show that the genetic program expressed in telia is more similar to the genetic program activated in uredinia than those observed in isolated resting urediniospores and during the biotrophic growth during poplar leaf infection, suggesting that overlapping sets of transcripts are important for both sporulation structures. Notably, we found at least two times more telia-specific genes compared to other investigated stages and most of them (69%) encode unknown proteins. Consistent with this, EST sequencing of pycniospores, aeciospores, teliospores, and urediniospores of the rust fungus *P. triticina* revealed that pycniospores and teliospores yield the largest sets of unique gene sequences (837 and 605, respectively), the majority of them (81 and 86%, respectively) having no functional annotation (Xu et al., 2011). Taken together, these results suggest that teliospore production involves largely unknown biological processes.

The telia structure plays a key role for spore survival over winter (Mendgen, 1984). Many transcripts are specifically expressed in telia or differentially up-regulated between telia and uredinia in *M. larici-populina* and they may be related to adaptation to cold temperatures and adverse winter conditions. Among these genes, several encode aquaporin water channels that may prevent osmotic damage of cells due to freezing. Previous studies have shown that aquaporins have desiccation and freeze tolerance functions in microorganisms, including bacteria, yeast, and fungi (Tanghe et al., 2006). Interestingly, one of the above-mentioned *M. larici-populina* aquaporin gene specifically expressed in telia (*Mlp-26257*) is orthologous to *AQY1*, a gene previously characterized together with *AQY2* in *Saccharomyces cerevisiae* and involved in a rapid, osmotically driven efflux of water during the freezing process that reduce intracellular ice crystal formation and resulting cell damage (Tanghe et al., 2002). Moreover, it has been also shown that AQY1 may also play a role in spore maturation in *S. cerevisiae* by allowing water outflow (Sidoux-Walter et al., 2004). We also identified three genes encoding osmotin/TLPs with a higher expression in telia than uredinia. In plants, osmotins belong the pathogenesis-related 5 family and have high sequence similarity with thaumatins that are sweet-tasting proteins (AnŽlovar and Dermastia, 2003). Genes encoding osmotins/TLPs are induced in plants in response to pathogens (Petre et al., 2011), cold (Kuwabara et al., 2002), drought (Jung et al., 2005), and osmotic stress (Singh et al., 1987). Induction of osmotins/TLPs during abiotic stress is often associated with osmotic adaptation in plant cells (Singh et al., 1987; Liu et al., 2010). Two osmotins/TLPs identify as highly expressed in *M. larici-populina* telia (*Mlp-76068, Mlp-79324)* correspond to small TLPs recently reported in basidiomycetes (Petre et al., 2011). These small TLPs belong to a monophyletic group with *Puccinia* TLPs indicating they may have evolved independently in pucciniales and plants (Petre et al., 2011). The role of TLPs in fungi has not been elucidated yet but our data suggest they might serve a possible role as an osmoprotectant in response to damaging effects of desiccation that can occur in teliospores during winter. Teliospores are highly melanized structures, and melanin is thought to provide protection against adverse environmental conditions. Genes encoding multi-copper oxidase laccase-like proteins, also identified in an EST library of *P. triticina* teliospores, are induced in telia and could be implicated in the biosynthesis of the melanin pigment (Xu et al., 2011). In basidiomycetes, recognition of mating partners is achieved through a pheromone/pheromone receptor system encoded by mating loci (Kronstad and Staben, 1997). In the smut fungus *Ustilago maydis*, the binding of pheromone to the receptor induces signaling cascades through specific mitogen-activated protein kinases pathways, and it is also marked in mating partners by the formation of conjugation tubes as well as G2 cell cycle arrest which ensure a synchronous stage of the cell cycle prior further developmental stages (Brefort et al., 2009). In the present case, we noticed that transcript levels from *M. larici-populina* mating loci genes, including pheromone receptor STE3 genes and a putative b mating loci gene are higher at late stages of plant colonization (i.e., formation of new urediniospores in the plant mesophyll) than in resting urediniospores or telia, although higher in telia than in resting urediniospores. A similar transcript profile is observed for the pheromone-regulated multispanning membrane protein Prm1 gene which is involved in plasma membrane fusion events during mating (Heiman and Walter, 2000). It is tempting to speculate that these mating-related genes could play a role in signaling during the rust fungus spore development and/or the control of cell cycle progression and cell fusion during formation of sporogenous hyphae and urediniospores.

Karyogamy and meiosis are crucial cellular processes that take place in teliospores. They play a fundamental role in generating genetic diversity by promoting recombination between chromosome homologs. In fungi, meiosis can drive genome plasticity and facilitates rapid adaptation to changing environments (Wittenberg et al., 2009; Goodwin et al., 2011) and it is a crucial process for pathogenic rust fungi to overcome R-gene mediated host disease resistance by diversification of virulence effectors. Karyogamy monitoring during teliospore formation and maturation revealed that teliospore initials are formed between poplar epidermal and palisade parenchyma cells around 16 dpi. DAPI-staining of teliospore nuclei also indicates that karyogamy is a dynamic process that mainly takes place between 25 and 39 dpi. Consistent with this, a previous study has shown that when *Populus tremula* leaves begin to wither, marginal teliospores of telia formed by *Melampsora pinitorqua* are in a dikaryotic stage whereas the more central ones are already in the diploid stage (Longo et al., 1979). *M. larici-populina* karyogamy-related genes *Kar5* and *Kar9* are both transiently induced during telial development from 25 dpi and their transcripts accumulate at 32 dpi for *Kar9* and 39 for *Kar5*, corroborating their implication in the karyogamy process. From 90 to 100% of the analyzed teliospores have fused nuclei at 39 and 46 dpi, respectively. As the microscopic observation of nuclei during karyogamy has been carried out only on a single biological replicate, we cannot exclude that slights variations may occur when analysing additional replicates. However, our results are consistent with previous results in *P. graminis* showing that all DAPI-stained teliospore protoplasts have fused nuclei at 42 dpi (Boehm et al., 1992). Several transcripts encoding meiosis-related genes are induced in *M. larici-populina* telia. RT-qPCR expression profiles of conserved meiosis-related genes during telia differentiation revealed transcripts accumulation between 39 and 46 dpi, indicating that meiosis occurs soon after karyogamy in the experimental conditions used in the study. This observation may differ under natural conditions with decreasing temperature during autumn. Consistent with the fact that meiosis is already initiated at 39 dpi, a spotty DAPI-staining was observed for most nuclei at that stage (data not shown). Among the conserved meiosis genes analyzed, Spo11 is a transesterase that creates DNA double strand breaks in homologous chromosomes (meiotic prophase 1, leptonema stage) (Keeney et al., 1997), Hop1 is a protein is required for synaptonemal complex formation (meiotic prophase 1, zygonema stage) (Aravind and Koonin, 1998), Mnd1 is a protein that ensure accurate and efficient meiotic interhomolog repair (meiotic prophase 1, pachynema stage) (Gerton and DeRisi, 2002) and REC8 is involved in sister chromatin cohesion (prophase 1) (Klein et al., 1999). These genes were identified as specifically expressed or differentially regulated in telia using oligoarrays and they are all involved in the early meiotic prophase stages (leptonema, zygonema, pachynema), supporting that meiosis is blocked in prophase I at the diplonema stage when teliospores enter dormancy (Boehm et al., 1992).

The accumulation of other transcripts may also reflect telia-specific features. For instance, a transcript encoding a saccharopine dehydrogenase in particular is specifically and highly expressed in telia. This gene belongs to the α-aminoadipate pathway that leads to the biosynthesis of the amino acid L-Lysine (Xu et al., 2006) and may play a crucial role in the overwintering structure. Several fungal alkaloids or peptides have lysine as a structural element or biosynthetic precursor and may accumulate in telia. Contrary to the uredinium that breaks through the epidermis to release large amounts of urediniospores (Hacquard et al., 2010), the telium is a structure that is encapsulated between poplar epidermis and palisade mesophyll cells that remains stable over the winter season. Expression of a cocktail of lytic enzymes that target the plant cell wall at early stages of telia development such as cellulases, hemicellulases, and pectinases might reflect accommodation of the telial structure to the decaying plant tissue.

To conclude, our transcriptomic analysis gives a first overview of the genetic program activated by rust fungi during telia formation. Particularly, we identified several genes encoding osmotins/thaumatin, aquaporin, and multi-copper oxidase laccase-like proteins that may reflect specific adaptation to cold environment and overwintering. Furthermore, our time course experiment study revealed the precise temporal dynamics of karyogamy and meiosis processes and suggests these are tightly regulated during teliospore formation and maturation.

## Author contribution

Stéphane Hacquard, Pascal Frey, and Sébastien Duplessis designed research; Stéphane Hacquard and Christine Delaruelle performed research; Stéphane Hacquard, Emilie Tisserant, and Annegret Kohler analyzed data; and Stéphane Hacquard, Pascal Frey, and Sébastien Duplessis wrote the paper.

### Conflict of interest statement

The authors declare that the research was conducted in the absence of any commercial or financial relationships that could be construed as a potential conflict of interest.

## Supplementary material

Figure S1 **Electrophoretic profiles of total RNA collected in the study**.

Table S1 **Summary of *M. larici-populina* genes selected for RT-qPCR analysis**.

Table S2 **Genome-wide expression analysis of *M. larici-populina* genes measured at the telial and uredinial stages using microarray**.
