# Supplementary material for: Transcriptome analysis of poplar rust telia reveals overwintering adaptation and tightly coordinated karyogamy and meiosis processes
Source: Front Plant Sci. 2013 Nov 21;4:456. doi: 10.3389/fpls.2013.00456 (PMC3835972; doi:10.3389/fpls.2013.00456)
Supplement: Figure S1 — Electrophoretic profiles of total RNA collected in the study. [file DataSheet1.ZIP › Table S1.docx]

**Table S1. Summary of *M. larici-populina* genes selected for RT-qPCR analysis**

| **Protein_ID^a^** | **Definition** | **Forward** | **Reverse** | **Amplicon size (bp)** | **Efficiency %** |
| --- | --- | --- | --- | --- | --- |
| 39329 | Double-strand break repair protein MRE11 | TTCGCCATAAATGCTGTTCC | GCGTGTATTCAACCCTCAATC | 247 | 100 |
| 49933 | DNA repair protein rad50 | GATGCTCAAATCGCTGGTCT | TGCAATCTCTCTGCATGGTT | 233 | 79 |
| 74723 | Septin homolog spn4 | TCAGAGTCCACGCCTGTCTT | CACCTCCCTGATACGTTGCT | 176 | 82 |
| 93153 | Meiotic recombination protein Rec8 | GCTTTCGATGTTGGAGAAGG | TACCGATCCACGTACCGATT | 174 | 104 |
| 94190 | Cell division control protein 15 | TTTCTCAGGCTCGAAGGAAG | CGGAGTGAATTTGTTTGTGG | 217 | 88 |
| 94206 | hypothetical protein related to KAR9 | CATTGTCCCGTTAGCTGGTT | CTGCTGAAGGTCCACCAAGT | 221 | 80 |
| 94329 | Cell division control protein 15 | AAAGGCGAGCATGAAGAGAC | TCGACCCTGCTTTGACTACC | 203 | 94 |
| 104525 | Meiotic rec protein, related to spo11 | TGAAGAAGACGTGCGATTTG | GAAGAAGTTGTCGGGTTGCT | 152 | 96 |
| 105250 | DNA repair protein RAD51 | CACAAAACCCACAAGCAGAA | TCCGAGTTGTATCTCTACCTTTCC | 246 | 105 |
| 106496 | MutS protein homolog 4 | CATCTCGGCTGGAGTAAAGG | CCAATCTGGTTTCGATGGTT | 166 | 101 |
| 106571 | Meiotic nuclear division protein 1 | TGATCGAGTGGAATCTGTTGA | TCCAATTCTCAGCACCTGTTT | 173 | 87 |
| 109837 | No hits found | TGGGAAACTGACGAGGAAAG | TTGTCGAGCAGCGAATATCA | 235 | 97 |
| 111529 | DNA mismatch repair protein Mlh1 | TTGTTGGTGTGGTGGATCTT | TTTCACTCGGTTCCCTCATC | 207 | 92 |
| 112713 | Nuclear Fusion protein KAR5 | TTTGACCACAGTTCCAACCA | AGAGCCAACCAGAGAAGCAG | 162 | 92 |
| 115763 | Cytokinesis protein sepA | CGTCGTGAATTGGCTTCTTT | GATCGCTTGGAAGGTGATGT | 158 | 93 |
| 116742 | MutS protein homolog 5 | AATAGAATGCCCGAAGACGA | CTCCAAACAAAGCTGCACAA | 236 | 102 |

^a^Protein ID number of corresponding best gene model in the *M. larici-populina* JGI genome sequence.
