# Supplementary figures and images for: Transcriptome analysis of poplar rust telia reveals overwintering adaptation and tightly coordinated karyogamy and meiosis processes
Source: Front Plant Sci. 2013 Nov 21;4:456. doi: 10.3389/fpls.2013.00456 (PMC3835972; doi:10.3389/fpls.2013.00456)

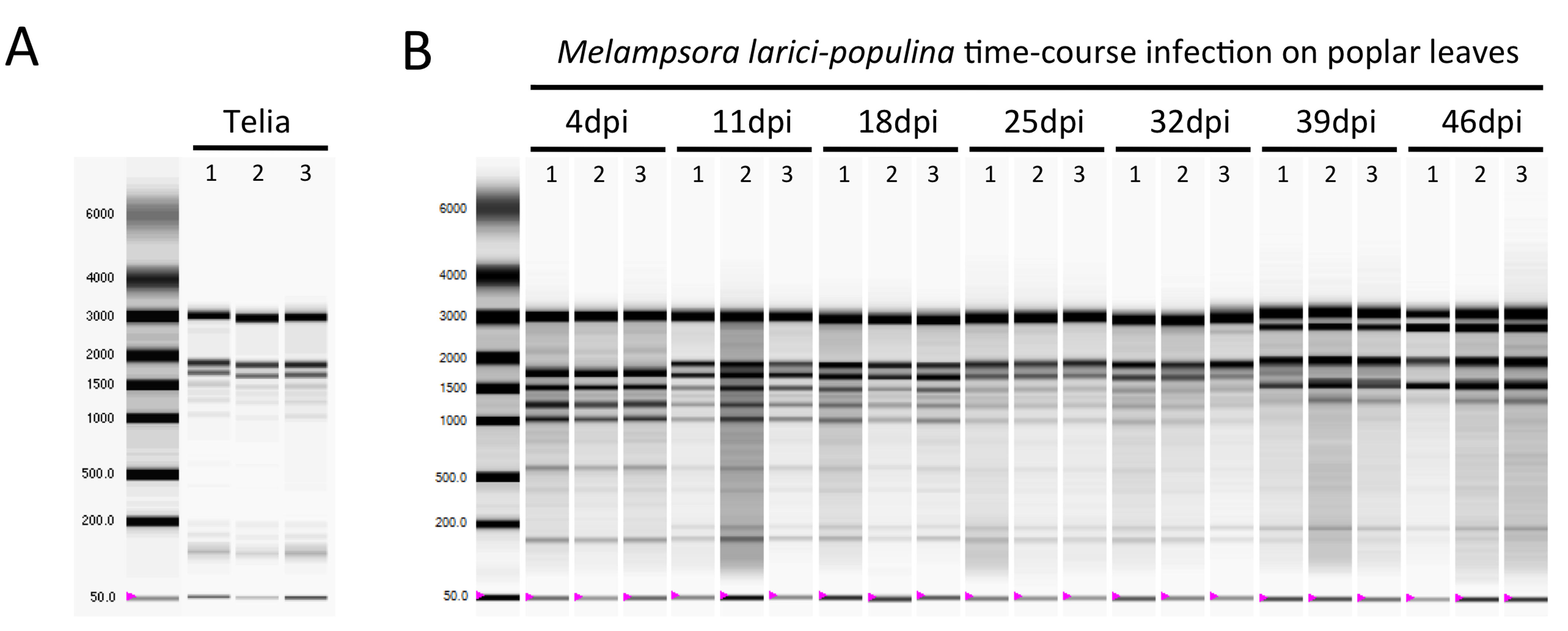

Supplement: Figure S1 — Electrophoretic profiles of total RNA collected in the study. [file DataSheet1.ZIP › Figure S1.tif]
